# Phylogenomic analysis shows underestimated species within *Cupriavidus* and the new species *Cupriavidus phytohabitans* sp. nov

**DOI:** 10.1038/s41598-026-39004-6

**Published:** 2026-02-13

**Authors:** Erika-Yanet Tapia-García, Belén Chávez-Ramírez, Leslie-Mariana Morales-Ruíz, Ivan Arroyo-Herrera, Violeta Larios-Serrato, J. Antonio Ibarra, Paulina Estrada-de los Santos

**Affiliations:** 1 Laboratorio de Biotecnología Microbiana, Departamento de Microbiología, Instituto Politécnico Nacional, Escuela Nacional de Ciencias Biológicas, Prol. Carpio y Plan de Ayala s/n. Col. Santo Tomás, Alcaldía Miguel Hidalgo, C.P. 11340 Mexico City, Mexico; 2 Departamento de Nutrición, Centro Interdisciplinario de Ciencias de la Salud, Instituto Politécnico Nacional, Unidad Milpa Alta, Ex Hacienda del Mayorazgo, km 39.5 Carretera Xochimilco – Oaxtepec, C.P. 12000 Mexico City, Mexico; 3 Laboratorio de Fitopatología, Departamento de Microbiología, Instituto Politécnico Nacional, Escuela Nacional de Ciencias Biológicas, Prol. Carpio y Plan de Ayala s/n. Col. Santo Tomás, Alcaldía Miguel Hidalgo, C.P. 11340 Mexico City, Mexico; 4 Laboratorio de Biotecnología y Bioinformática Genómica, Departamento de Bioquímica, Instituto Politécnico Nacional, Escuela Nacional de Ciencias Biológicas, Prol. Carpio y Plan de Ayala s/n. Col. Santo Tomás, Alcaldía Miguel Hidalgo, C.P. 11340 Mexico City, Mexico; 5 Laboratorio de Genética Microbiana, Departamento de Microbiología, Instituto Politécnico Nacional, Escuela Nacional de Ciencias Biológicas, , Prol. Carpio y Plan de Ayala s/n. Col. Santo Tomás, Alcaldía Miguel Hidalgo, C.P. 11340 Mexico City, Mexico

**Keywords:** *Cupriavidus*, Nodule-associated bacteria, Nodulation, *Phaseolus vulgaris*, Rhizosphere, Trap plant, Genetics, Microbiology, Plant sciences

## Abstract

During a survey of nodule-associated bacteria from legume plants and soil collected in southern Mexico, several strains were obtained in Mexico City using *Phaseolus vulgaris* as a trap plant, inoculated with rhizospheric soil from the legume *Acacia* sp. collected in the state of Veracruz, a bordering state to the Gulf of Mexico. The 16S rRNA gene sequence analysis identified the strains AcVe19-1a^T^, AcVe19-6a, and AcVe19-6b as *Cupriavidus* (with 99.9% similarity to many type strains of *Cupriavidus* species and 99.9 to 100% similarity within the novel species). Comparative genomics by average nucleotide identity (ANI) and digital DNA-DNA hybridization (dDDH) showed that strains AcVe19-1a^T^ and AcVe19-6a correspond to a new *Cupriavidus* genomic species (ANI values lower than 94% and dDDH values lower than 55% to any *Cupriavidus* strain, and the closest species *Cupriavidus consociatus*). The genome of the novel species harbors nitrogen-fixing and nodulation genes, enabling it to nodulate plants of *P. vulgaris* and *Mimosa pudica,* but it does so poorly with *Acacia* sp. However, nitrogen was not fixed in the nodule or the culture medium. According to the symbiovars guidelines, two symbiovars are proposed. The genome analysis, together with phenotypic, physiological, and chemotaxonomical analyses, corroborate that strains AcVe19-1a^T^, AcVe19-6a, and AcVe19-6b correspond to a novel species, for which we propose the name *Cupriavidus phytohabitans* sp. nov. with the type strain AcVe19-1a^T^ = TSD-313^T^ = CDBB B-2084^T^. In addition to the description of the novel species, a comparative genomic analysis of all *Cupriavidus* species in the NCBI database showed that many have been misclassified, and others correspond to new genomic species.

**Supplementary Information:**

The online version contains supplementary material available at 10.1038/s41598-026-39004-6.

## Introduction

The genus *Cupriavidus* belongs to the phylum Pseudomonadata, the class Betaproteobacteria, the order Burkholderiales, and the family Burkholderiaceae. Currently, the genus *Cupriavidus* comprises 23 species, with *Cupriavidus necator* as the type species. Three species names have not been validly published under the International Code of Nomenclature of Prokaryotes (ICNP) in the List of Prokaryotic names with Standing in Nomenclature (https://www.bacterio.net/), which are “*C. eutrophus", “C. malaysiensis”,* and “*C. neocaledonicus*". Other new *Cupriavidus* species are awaiting publication, such as "*C. gehlotti"* and "*C. mimosae"* ^1^, and "*C. ariensis*" (Raúl Platero, Instituto de Investigaciones Biológicas Clemente Estable, Uruguay, personal communication). There are also some *Cupriavidus* species able to nodulate legume plants, such as *C. taiwanensis* (effective nodules on *Mimosa* species)^2^, *C. necator* (effective nodules on *Mimosa caesalpiniaefolia*, *Leucaena leucocephala*, *Macroptilium atropurpureum*, *Phaseolus vulgaris*, and *Vigna unguiculata*)^3^, “*C. neocaledonicus*” (effective nodules on *Mimosa pudica*)^4^, *C. phytorum* (only strain LMG 19430 was able to produce effective nodules on *M. pudica*)^5^, some *Cupriavidus* sp. strains isolated from plants from *Parapiptadenia rigida* (effective nodules on *Mimosa*)^6^, and few *Cupriavidus* sp. strains isolated from *Mimosa magentea*, *Mimosa ramulosa*, *Mimosa schleidenii*, *Mimosa reptans* and *Mimosa cruenta* (effective nodulation in *M. pudica*, *Mimosa polycarpa*, *M. magentea*, *M. ramulosa* and *M. schleidenii*)^7^. Recent studies of nodule-associated bacteria in Mexico have revealed the presence of *Cupriavidus* sp. in Chiapas, located in southern Mexico, as well as in nodules from *P. vulgaris* plants inoculated with soil obtained in Veracruz, a state situated on the Gulf of Mexico^8^. Among these strains, two novel species were recently proposed: *C. phytorum,* isolated from *Zea mays* rhizosphere and *Mimosa diplotricha* root nodules^5^, and *C. consociatus,* isolated from *Arachis* sp. and *Leucaena* sp. plant nodules^9^. The type strain of *C. phytorum* cannot fix nitrogen or nodulate, and no information on either activity is found in its genome sequence. Still, the reference strain *C. phytorum* LMG 19,430 can, as previously described^9^. *C. consociatus* contained nitrogen fixation and nodulation information in its genome sequences; however, no nitrogen fixation was detected in the culture medium or nodules.

The presence of *Cupriavidus* in Mexico extends beyond its association with legumes, including agave, maize, sugarcane, and sorghum^10^. Three *Cupriavidus* species have been described from the previous plants, namely *C. alkaliphilus*, *C. plantarum,* and *C. agave*, in the northeast of Mexico^11–13^.

In this study, we continue the characterization of nodule-associated *Cupriavidus* strains isolated from *P. vulgaris* nodules used as trap plants inoculated with the rhizospheric soil from *Acacia* sp. growing in the wild in Veracruz, a state bordering the Gulf of Mexico. Moreover, a massive comparative genomic analysis revealed that *Cupriavidus* contains many more species than are presently described.

## Results and discussion

### 16S rRNA gene sequence analysis

One of the first steps in identifying bacterial species is 16S rRNA gene sequence analysis, considering a threshold of approximately 98.7% used to delineate a new species^14^. However, at present, given the high similarity in 16S rRNA gene sequences among bacterial species, the analysis of the gene serves only as a reference to infer a strain’s position at the genus or higher taxonomic level^15^. In this study, the 16S rRNA gene sequence similarity among AcVe19-1a^T^, AcVe19-6a, and AcVe19-6b strains was 99.9 – 100%. The 16S rRNA gene sequence analysis showed that the three strains belonged to the genus *Cupriavidus,* with 99.9% similarity to many type strains of *Cupriavidus* species. The phylogenetic analysis revealed that the new species was close to *C. taiwanensis* and *C. nantongensis* (Fig. S1), corroborating the identification of the strains as members of the genus *Cupriavidus*. By comparative genomics (as shown in the following section), the new species also included *Cupriavidus* sp. AMP6, which also clusters with the new species using the 16S rRNA gene sequence analysis.

### Genome features

To obtain a more accurate description of a new bacterial species, the genome sequence is currently required. The features encoded in the genome and obtained after genome assembly and annotation are also essential. Therefore, in this study, the genome sequence of strain AcVe19-1a^T^ was assembled in 210 contigs, with a genome length of 7,423,665 bp. The number of contigs for the genome of strain AcVe19-6a was 315, with a genome length of 7,432,279 bp. The G + C content was 65.7 and 65.58% mol for strains AcVe19-1a^T^ and AcVe19-6a, respectively. More information about the genomes of strains AcVe19-1a^T^ and AcVe19-6a is presented in Table S1, where the data are very similar between the two strains. Moreover, *Cupriavidus* sp. AMP6, according to the NCBI, was assembled into 264 contigs, with a total genome length of 7.6 Mb and a G + C content of 65.5%mol, similar to strains AcVe19-1a^T^ and AcVe19-6a.

### Measurements of genomic relatedness

**Fig. 1:**
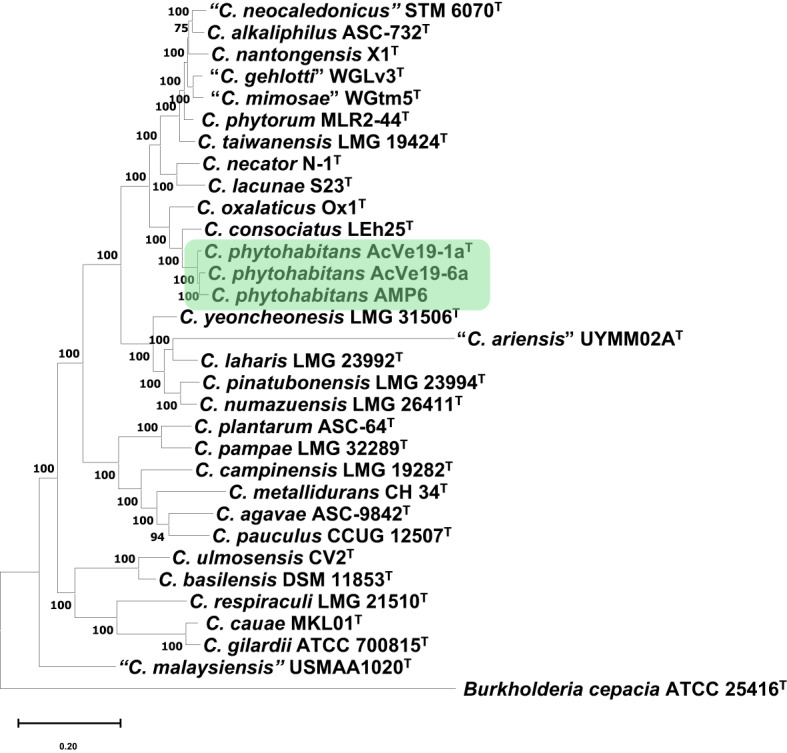
Phylogenomic analysis of type strains of *Cupriavidus* species. The analysis was performed with 400 conserved universal markers selected by PhyloPhlAn for deep-branching phylogenies. The novel species is pointed out in green. The numbers in the branches correspond to a 1000-bootstrap analysis. The bar corresponds to the number of differences between the sequences.

The overall genomic relatedness indices (OGRIs), calculated using several methods, are currently standard for delineating a new prokaryotic species^15^. Two indices are typically used to define a genomic species: average nucleotide identity (ANI) and digital DNA-DNA hybridization (dDDH)^16,17^. The values to define a new species are below 95–96% for ANI and 70% for dDDH. Accordingly, in this study, a genomic comparison of the novel species was conducted with all type and reference strains of *Cupriavidus* species and *Cupriavidus* sp. strains (meaning strains not assigned to any *Cupriavidus* species), revealing that *Cupriavidus* sp. AMP6 also belongs to the novel species (Table 1). *Cupriavidus* sp. AMP6 was isolated from root nodules of *Mimosa asperata* growing in Santa Ana National Wildlife Refuge, Texas, U.S., in 2005^18^, similarly to the niche where strains AcVe19-1a^T^, AcVe19-6a, and AcVe19-6b were isolated, i.e., nodules of *P. vulgaris*. The intra-species similarity values as determined by ANI and dDDH for the novel genomic species (including strain AMP6) were 99.05 – 99.18% and 91.1 – 92.9%, respectively, placing them within a single *Cupriavidus* genomic species. The closest species were *C. consociatus* and *C. oxalaticus,* with values below the threshold for delineating new species (Table 1). The genome comparison with other type strains of *Cupriavidus* species and *Cupriavidus* sp. showed ANI and dDDH values below 85% and 40%, respectively. The phylogenomic analysis clustered AcVe19-1a^T^, AcVe19-6a, and AMP6 close to *C. consociatus* LEh25^T^ and *C. oxalaticus* Ox1^T^ (Fig. 1). The genomic measurements confirm that strains AcVe19-1a^T^, AcVe19-6a, and AMP6 correspond to a new genomic species within the genus *Cupriavidus*.

**Table 1:** Comparative genomics by average nucleotide identity and digital DNA-DNA among *Cupriavidus phytohabitans* sp. nov. and the closest type strains of *Cupriavidus* species.

*Cupriavidus* species	ANI			dDDH		
	AcVe19-1a^T^	AcVe19-6a	AMP6	AcVe19-1a^T^	AcVe19-6a	AMP6
*C. phytohabitans* AcVe19-6a	**99.18**	–	–	**92.9**	–	–
*Cupriavidus* sp. AMP6	**99.05**	**99.01**	–	**91.6**	**91.1**	–
*C. consociatus* LEh25^T^	93.52	93.60	93.51	53.6	53.8	53.5
*C. oxalaticus* Ox^T^	93.43	93.62	93.32	53.6	53.8	52.9

ANI, average nucleotide identity, was calculated using FastANIb with anvi’o^40^. dDDH, digital DNA-DNA, was performed using the Genome-to-Genome Distance Calculator (https://ggdc.dsmz.de/home.php). In bold are the values that validate the new species.

### Morphology, physiology, and biochemical analysis

Although phenotypic analyses are not definitive in distinguishing a new species from others, providing as much information as possible when a new taxon is proposed is essential. Consequently, the phenotypes of strains AcVe19-1a^T^, AcVe19-6a, and AcVe19-6b were analyzed, revealing that the cells stain Gram-negative and the rods are 1.3 μm long and 0.5 μm wide (Fig. S2). The colonies were circular, convex, glossy, and beige-colored in LB medium. The strains grew on LB agar at temperatures ranging from 20 to 37 °C, and on YM and MacConkey agar at temperatures ranging from 30 to 37 °C. Growth at 42 °C was strain-dependent (Table S2). They grew on modified LB up to 1% NaCl, but AcVe19-1a^T^ up to 2% NaCl. The pH range for growth was 6.0 to 9.0; however, AcVe19-1a^T^ grew between pH 5.0 and 9.0. The alkalinization of L-lactate and succinate was positive. The activity of gamma-glutamyl-transferase, L-proline-arylamidase, tyrosine arylamidase, and phosphatase was positive. The strains assimilate citrate, malonate, L-malate, Ellman, and L-lactate. The complete results from biochemical and phenotypic analyses are presented in Table S2, and differential features relative to the closest and most relevant species are shown in Table 2.

**Table 2:** Distinctive phenotypic features among *Cupriavidus phytohabitans* sp. nov. and the closest and relevant *Cupriavidus* species.

Phenotypic feature	*Cupriavidus phytohabitans* AcVe19-1a	*Cupriavidus phytohabitans* AcVe19-6a	*Cupriavidus phytohabitans* AcVe19-6b	*Cupriavidus consociatus* LEh25^T^	*Cupriavidus oxalaticus* Ox1^T^	*Cupriavidus necator* N-1^T^	*Cupriavidus taiwanensis* LMG 19424^T^
Isolation source	*Phaseolus vulgaris* nodules	*Phaseolus vulgaris* nodules	*Phaseolus vulgaris * nodules	*Leucaena* sp. nodules	Alimentary tract earthworm	Soil	*Mimosa pudica* nodules
Location source	Veracruz, México	Veracruz, México	Veracruz, México	Chiapas, México	India	Pennsylvania, United States	Ping-Tung Country, Taiwan
Growth on LB* agar + NaCl (%):							
2.0	+	−	−	+	+	+	+
3.0	−	−	−	−	−	−	+
Growth at pH values:	5.0 – 9.0	6.0 – 9.0	6.0 – 9.0	6.0 – 7.0	8.0 − 9.0	6.0 – 9.0	6.0 – 9.0
Activity of:							
L-Pyrrolydonyl-arylamidase	−	−	−	−	−	+	+
Glutamyl arylamidase pNA	−	−	−	−	−	+	+
Gamma-glutamyl-transferase	+	+	+	+	−	+	+
Urease	−	−	−	−	−	−	+
Phosphatase	+	+	+	−	+	+	+
Glycine arylamidase	−	−	−	−	−	−	+
Assimilation of:							
Malonate	+	+	+	+	−	−	−
L-histidine	−	+	−	−	−	−	−
L-malate	+	+	+	+	−	+	+
L-lactacte	+	+	+	+	−	+	+

*LB modified, without NaCl.

### Chemotaxonomic analysis

Chemotaxonomy is also included in phenotypic tests to characterize a new bacterial species. For example, whole-cell protein patterns obtained by highly standardized SDS-PAGE have been proven to be extremely reliable for comparing and grouping closely related strains^19^. Its use for general identification purposes yields discriminative information at or below the species level^19^. These patterns were analyzed in strains AcVe19-1a^T^, AcVe19-6a, AcVe19-6b, *C. consociatus* LEh25^T^, and *C. oxalaticus* Ox1^T^. The protein patterns from strains AcVe19-1a^T^ and AcVe19-6a are identical and highly similar to strain AcVe19-6b but different from *C. consociatus* and *C. oxalaticus* (Fig. S3), which was the expected result. Another chemotaxonomic analysis involves determining the total polar lipids, which are the major constituents of the lipid bilayer in bacterial membranes and have been studied for classification and identification purposes. The polar lipids in strain AcVe19-1a^T^ consisted of phosphatidylethanolamine (PE), diphosphatidylglycerol (DPG), phosphatidylglycerol (PG), cardiolipin (CL), and an unknown aminolipid, which are different from the closest species *C. consociatus* and *C. oxalaticus* (Fig. S4).

### Phylogenetic analysis of ubiquinones

**Fig. 2:**
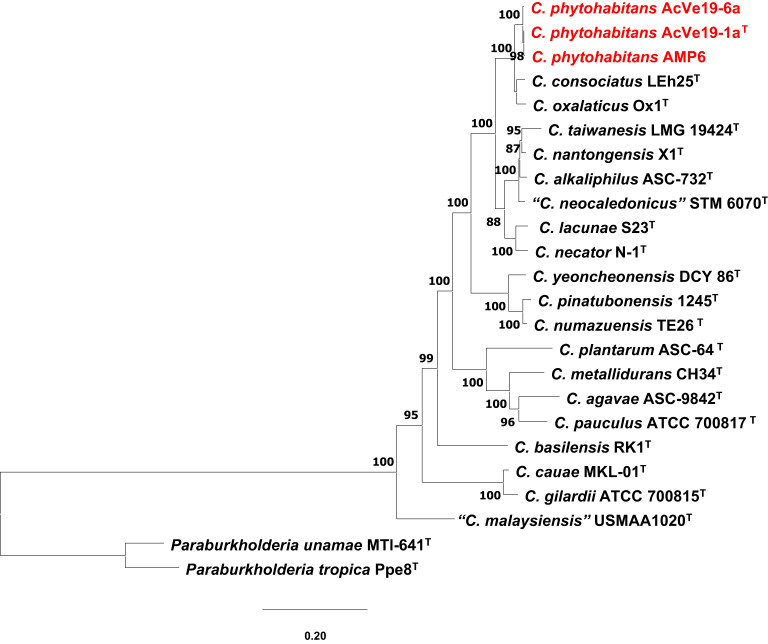
Phylogenetic analysis of concatenated ubiquinone amino acid sequences from type strains of *Cupriavidus* species. The inference used the maximum-likelihood method and the amino acid substitution model BLOSUM62. The number on the branches represents bootstrap support generated from 1000 replicates. Bar, 0.2 nucleotide substitutions per position. The novel species is highlighted in red.

In most living organisms, catalytic reactions involved in cellular energy generate electrons, which are channeled to the quinone pool. Then, the reduced quinones serve as substrates for reducing the terminal acceptors. Microorganisms contain various quinones, including ubiquinone, also known as coenzyme Q (Q), menaquinone (MK), and dimethylmenaquinone (DMK). Ubiquinones can be determined in the laboratory or analyzed phylogenetically as well. *Cupriavidus* species contain ubiquinone, and the amino acid sequences from UbiA, UbiB, UbiD, UbiE, UbiF, UbiG, UbiH, UbiI, UbiJ, and UbiX were obtained from all type strains of *Cupriavidus* species genomes and compared to those of the novel species. The phylogenetic analysis, comprising 3897 positions, revealed that the three strains encompassing the new species clustered together, close to *C. consociatus* and *C. oxalaticus* (Fig. 2). Nine *Cupriavidus* species have been reported to contain Q-8 as the major isoprenoid quinone. Given the conserved ubiquinone sequences among the *Cupriavidus* genus, it suggests that all *Cupriavidus* species may contain ubiquinone Q-8, including the novel species *C. phytohabitans*.

### Resistome analysis

Currently, there is a significant concern about antimicrobial resistance. It has been predicted that by 2050, there will be approximately 10 million deaths per year^20^. That is why elucidating the resistome of new bacteria is essential. Although the genus *Cupriavidus* is not widely recognized as a pathogen, certain strains of *C. gilardii, C. taiwanensis*, or *C. pauculus* have been associated with human infections^21–23^. The resistome analysis showed that the novel species is predicted to be resistant to tetracycline and fluoroquinolone antibiotics. Moreover, a genome analysis of 97 *Cupriavidus* strains identified 12 significant antibiotic resistance genes, all of which are associated with five efflux pumps^23^. Thus, the potential resistance of *Cupriavidus* should be taken seriously, especially since some species can behave as opportunistic pathogens.

### Nitrogen fixation and nodulation analysis

Nitrogen fixation is crucial in the nitrogen biogeochemical cycle and is essential for plant growth and development. Bacteria perform nitrogen fixation either freely or in symbiosis with legume plants, utilizing the enzyme nitrogenase, which is encoded by several genes^24^. Since the novel species was isolated from root nodules, they were tested for nitrogen fixation in culture medium, yielding a negative result. However, to confirm that the test was conducted adequately, *Azospirillum brasilense* Sp7 and *Paraburkholderia tropica* Ppe8^T^, widely known as free nitrogen fixers, were used as controls. The analysis showed that both fix nitrogen in the culture medium. Many nodulating bacteria are unable to fix nitrogen in culture medium; however, it has been demonstrated that *Paraburkholderia phytmatum* STM 815^T^ can fix nitrogen when a small amount of yeast extract is added to the culture medium^25^. Nevertheless, this was not the case for the novel species, as it also happened with *C. taiwanensis* LMG 19424^T^^25^.

**Fig. 3:**
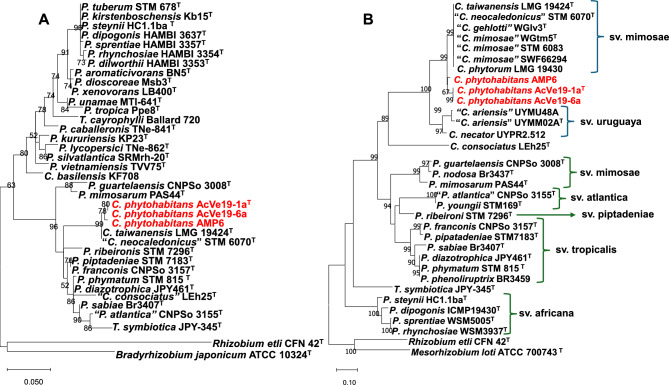
Phylogenetic analysis of NifH and NodC amino acidic sequences from *Cupriavidus* species and other nitrogen-fixing and nodulating bacteria. (**A**) NifH corresponds to nitrogen fixation activity. (**B**) NodC corresponds to nodulation activity. In brackets and arrows, the symbiovars from *Cupriavidus* and *Paraburkholderia* are indicated. The phylogenetic analysis was performed with the maximum-likelihood method and the amino acid substitution model BLOSUM62. The numbers at branch points correspond to bootstrap support generated from 1000 replicates. Bar, number of nucleotide substitutions per position.

**Fig. 4:**
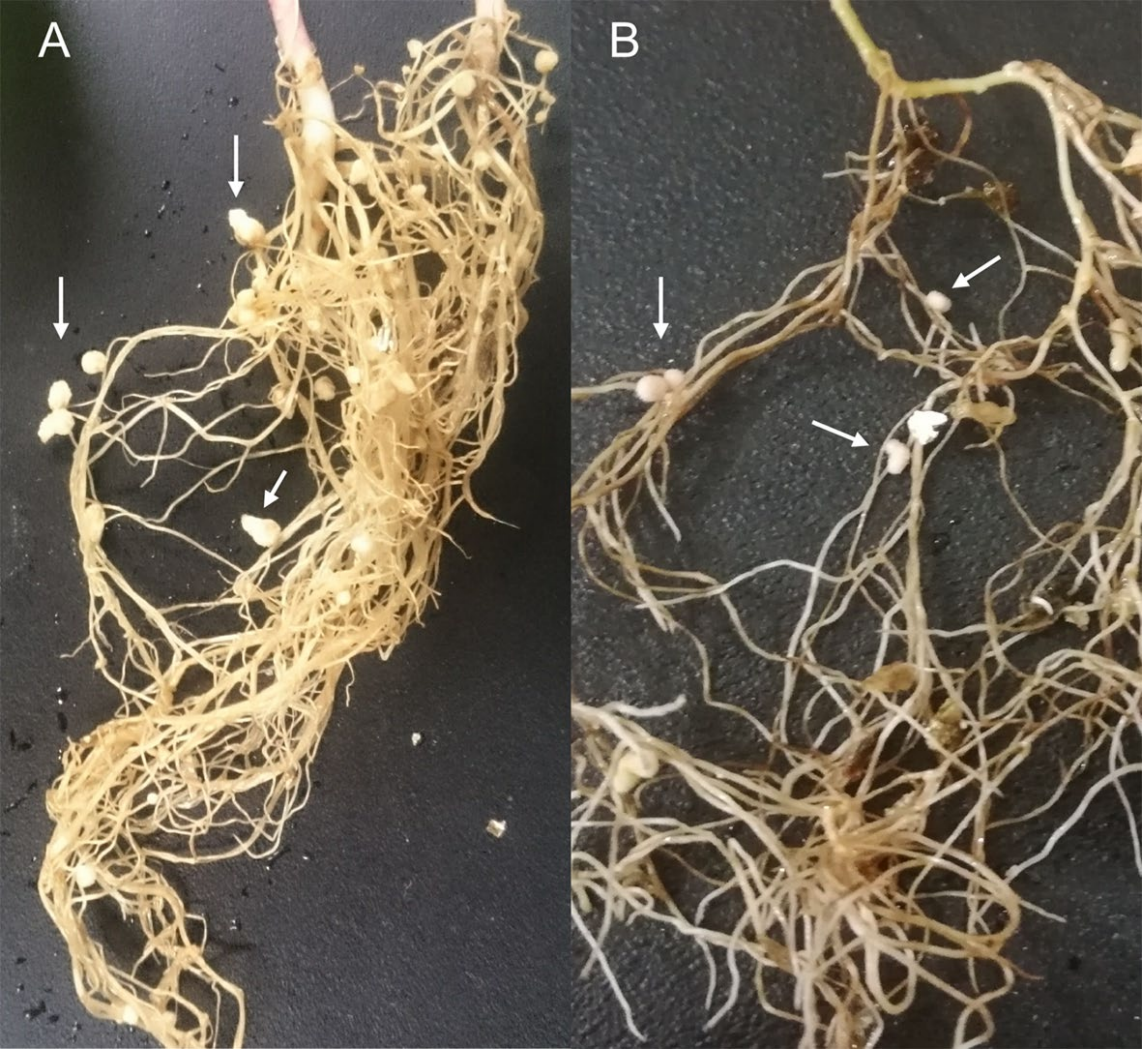
Nodulation activity of *Cupriavidus phytohabitans* sp. nov. (**A**) *Phaseolus vulgaris* inoculated with strain AcVe19-1a^T^. (**B**) *Mimosa pudica* inoculated with strain AcVe19-6a. The arrows indicate white, ineffective nodules.

The gene *nifH*, one of the most frequently used for phylogenetic comparison among nitrogen-fixing bacteria, was compared between the novel species and many nitrogen-fixing bacteria. The phylogenetic analysis of the *nifH* gene showed that the novel species cluster together, close to *C. taiwanensis* and “*C. neocaledonicus*”, and all of them are immersed among *Paraburkholderia* species (Fig. 3A). Nitrogen fixation can also occur in a symbiosis between a nitrogen-fixing bacterium and a legume plant, where root nodules are formed. Nodule formation requires that the bacterium express the *nod* genes, among others. The novel species was inoculated in *P. vulgaris* and *M. pudica* plants. Strain AcVe19-1a^T^ formed white ineffective nodules in all *P. vulgaris* plants; no nodulation was observed in *M. pudica*. Strain AcVe19-6a formed ineffective nodules in all *M*. *pudica* plants; no nodules were observed in *P. vulgaris* (Fig. 4). The number of nodules was more than ten in *P. vulgaris* and up to ten nodules in *M. pudica* (Fig. 4). The strain AMP6, isolated from root nodules of *M. asperata,* is capable of forming effective nodules on *Mimosa pigra, M. pudica,* and *Mimosa invisia* and ineffective nodules on *Mimosa strigillosa,* although it has not been tested in *M. asperata*, the original host to this strain^18^. The control bacteria used in the experiment were *Rhizobium* sp. MpTa5-8a and AcVe20-20b, which formed effective nodules in *P. vulgaris*. The control plants inoculated with water did not show any nodules, indicating the absence of contamination. Moreover, a nodulation test was performed on *Acacia* sp. with the novel species, but the results were inconclusive. Only one or two tiny nodules were observed, even after the test was repeated twice. This suggests that *Acacia* sp. may not be the original host of the new species, even though they were isolated from nodules of *P. vulgaris* used as a trap plant inoculated with rhizospheric soil from *Acacia* sp.

The inability of the novel species to fix nitrogen in the nodules of *Acacia* sp., *P. vulgaris,* and *M. pudica* shows that there appear to be impediments to perform this activity, despite nodules being well formed in *P. vulgaris* and *M. pudica,* but white instead of the expected pink color (Fig. 4). Some years ago, *Cupriavidus* strains were isolated from *Mimosa asperata* and *Mimosa strigillosa,* classified as related plant species^18^. Some of these strains contain highly similar *nodA* and *nifH* gene sequences; however, they nodulate *M. pigra*, *M. pudica*, *M. invisa*, *M. strigillosa,* and *M. quadrivalvis* differentially. Moreover, the strains isolated from *M. strigillosa* were unable to form nodules on this species, leaving open the question of which plant is the original host. The lack of nitrogen fixation in nodules was also observed with *C. consociatus*, a species isolated from *Leucaena* sp. and *Arachis* sp. that was unable to fix nitrogen in the nodules of *P. vulgaris* and *Leucaena* sp.^9^.

Symbiosis is a complex and structured process in which the host and the bacterial strain contribute to the establishment of root nodules. This opens a new line of study to determine the causes for the novel species forming few ineffective nodules in *Acacia* sp. and being unable to fix nitrogen in other plant nodules. To gain a deeper understanding of the previous situation, an analysis of nodulation and nitrogen fixation information in the novel species’ genome sequences was conducted. The *nod, nol, noe, nif, fix,* and *fdx* genes were extracted from the genome of strains AcVe19-1a^T^ (access number at JGI-DOE, USA 2909004890), AcVe19-6b (access number 2909011937), AMP6 (access number 2524023212), *C. taiwanensis* LMG 19424^T^ (access number 644736347) as the first effective nodulating bacteria in the genus *Cupriavidus*, *C. consociatus* LEh25^T^ (access number 2909019044) as another non-effective nodulating species, *P. phymatum* STM 815^T^ (access number 642555112) as a close genus from the betaprotebacteria, and *Bradyrhizobium* sp. DOA9 (accession 2579778901) as a distant nodulating bacterium. The amino acid information was checked for potential stop codons or insertions, but none were found in the novel species. Nodulation and nitrogen fixation genes from strain AcVe19-1a^T^ were compared to the list of strains mentioned above (Table S3). Strains AcVe19-1a^T^ and AcVe19-6a shared the same nodulation and nitrogen fixation information and organization, and this was generally 100% identical. There were differences with strain AMP6, but the protein identity with other species, such as *C. taiwanensis* LMG 19424^T^, *C. consociatus* LEh25^T^, and *P. phymatum* STM815^T^, was lower. The lowest identity was found with *Bradyrhizobium* sp. DOA9.

It was also found that not all *nod, nol, noe, nif, fix,* and *fdx* genes formed a single operon (Fig. S5). Given that the genomes are fragmented and gene information is distributed across different scaffolds, it is difficult to determine the exact gene organization and orientation. It was also found that some genes were missing in the new species, such as *nodZ, nolK, nolY, noeE, noeI, nifZ, fixQ, fixH, fixS, fixF*, and *fdxB* (Table S3), which may explain the lack of nitrogen fixation in the nodule and in culture medium. However, this may not be certain, as potential sequencing errors may lead to incomplete information. However, some of the previous genes, except DO09, were absent in the new species and the symbiotic species (*nodZ, nolY, noeE, noeI, fixF,* and *fdxB*) (Table S3), suggesting that these genes might not be necessary for nitrogen fixation in the nodule for the symbiotic species and therefore not an impediment for the novel species to fix nitrogen in the nodule. However, strains AcVe19-1a^T^ and AcVe19-6b lack the *nifZ* gene, which is a nitrogenase MoFe maturation protein, whereas strain AMP6 does not and can fix nitrogen in the nodule, as do *C. taiwanensis* LMG 19424^T^ and *P. phymatum* STM 815^T^. Therefore, *nifZ* in the new species may play an essential role in nitrogen fixation within the nodule.

Thus, these analyses suggest that further investigation is necessary to understand the mechanisms of nodulation and nitrogen fixation in the novel species.

The *nodC*, the best representative of *nod* genes, was compared among the strains, showing that the novel species cluster with each other, and close to a group containing *C. taiwanensis*, “*C. neocaledonicus*”, “*C. gehlotti*”, “*C. mimosae*”, and *C. phytorum* (Fig. 3B). Some years ago, the term symbiovar (symbiotic variant) was proposed by Rogel et al.^26^, defining the symbiotic capabilities of bacteria to nodulate host plants. However, due to legume promiscuity by nodulating bacteria, the identification of symbiovar was proposed to be based on the analysis of symbiotic genes, such as *nodC* or *nodA*^27^. However, recently, some new guidelines were proposed for a more accurate description of rhizobial symbiovars, summarizing the following description: a) plant specificity combined with effective nodulation, b) the genome of a representative strain must be available, c) symbiosis genes must be distinct from all previously described symbiovars^28^.

Currently, no symbiovars have been proposed for the nodulating *Cupriavidus* species. Following the guidelines and according to the phylogenetic analysis of *nodC* gene, four groups of nodulating *Cupriavidus* were formed. Thus, we propose the following symbiovars: symbiovar mimosae for the group containing *C. taiwanensis,* the first species isolated from *M. pudica,* and *M. diplotricha*, which is also capable of effectively nodulating both *Mimosa* species^2^. Symbiovar mimosae also contains “*C. neocaledonicus*”, “*C. gehlotti*”, “*C. mimosae*”, and *C. phytorum* (Fig. 3B).

A second symbiovar corresponds to symbiovar uruguaya, which is formed by the species “*C. ariensis*” isolated from *Mimosa magentae*, where it also exhibits effective nodulation^7^.

There are two other groups of *Cupriavidus* species: one containing the novel species *C. phytohabitans* and the second, *C. consociatus*; however, these species form ineffective nodules with their host plants and therefore do not fulfill guideline a mentioned above.

Moreover, *Paraburkholderia* species selected for *nodC* comparative analysis cluster with the corresponding symbiovars described by Paulistich et al.^27^, including symbiovars mimosae, atlantica, piptadeniae, tropicalis, and africana, all of which differ from the proposed symbiovars in *Cupriavidus* (Fig. 3B).

### Comparative genomic analyses of *Cupriavidus*

**Fig. 5:**
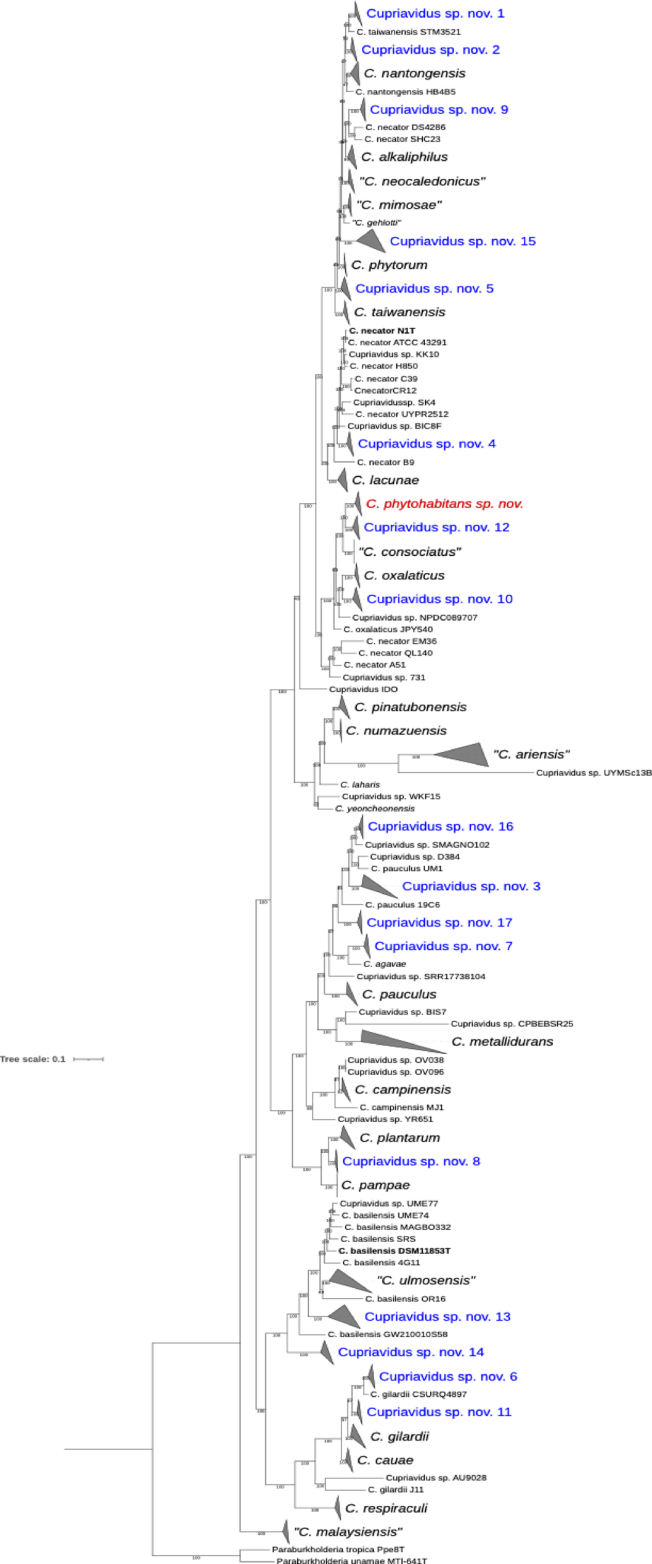
Phylogenomic analysis of type and reference strains of *Cupriavidus* species and *Cupriavidus* sp. The study was performed with 400 conserved universal markers selected by PhyloPhlAn for deep-branching phylogenies. In red is indicated *Cupriavidus phytohabitans* sp. nov., and in blue are pointed out the new species within *Cupriavidus*. The numbers in the branches correspond to a 1000-bootstrap analysis. The bar corresponds to the number of differences between the sequences.

All *Cupriavidus* strain genome sequences available in the NCBI database were downloaded and compared using ANI. The entire results are shown in Fig. S6, but an extract of the information and additional analysis using dDDH with GGDC are displayed in Table 3. Both analyses and a phylogenomic study (Fig. 5) revealed that many strains have been misclassified, particularly those from the *C. taiwanensis*, *C. necator,* and *C. gilardii* species. The results are described in the next section, where we found that, in a few cases, strains with an ANI of 95–96% had a dDDH below 70%. Therefore, we decided to establish the cut-off at dDDH 70% to define a new species or to include a new strain in an already described species. Recently, Chen et al.^23^ performed a genomic comparison of 97 *Cupriavidus* strains. We will compare their results with ours when appropriate. It is also important to mention that we compared each *Cupriavidus* species with its type strain.

**Table 3:** Comparative genome analysis of *Cupriavidus* species by digital DNA-DNA and average nucleotide identity.

Species	dDDH	ANI	Species	dDDH	
1. *Cupriavidus alkaliphilus*	ASC-732^T^		22. *Cupriavidus respiraculi*	LMG 21510^T^	
			*C. respiraculi* LMG 21510^T^	100	100
*C. alkaliphilus* ASC732^T^	100	100	*C. respiraculi* AU7532	90.3	98.7
*C. alkaliphilus* ASC-869	84	98	*C. respiraculi* CGMCC116115	99.6	99.9
*C. alkaliphilus* CAG-122	94.9	99.1	*C. respiraculi* CIP 108131^T^	100	100
*C. alkaliphilus* EM22	93.4	99.1	23. *Cupriavidus taiwanensis*	LMG 19424^T^	
*C. alkaliphilus* EM23	93.4	99.1	*C. taiwanensis* LMG 19424^T^	100	100
*C. alkaliphilus* MtRBr-3211	86.8	98	*C. taiwanensis* LMG 19426	91.4	98.8
*C. alkaliphilus* SLV-2362	93.4	99.1	*C. taiwanensis* LMG 19431	91.4	98.9
*C. alkaliphilus* SrBRr-232	83.4	98	*C. taiwanensis* SMAGNO117	90.7	98.8
*C. taiwanensis* cmp52	71.52	96.8	*C. taiwanensis* STM6018	91.5	98.8
2.* “Cupriavidus ariensis”*	UYMMa02A^T^		*C. taiwanensis* STM6021	90.7	98.7
*Cupriavidus* sp. UYMMa02a^T^	100	100	*C. taiwanensis* STM6032	91.5	98.9
*Cupriavidus* sp. UYMU48A	79.9	97.1	*C. taiwanensis* STM6041	90.8	98.8
*Cupriavidus basilensis*	DSM 11853^T^		*C. taiwanensis* STM6043	90.7	98.7
*C. basilensis* DSM 11853^T^	100	100	*C. taiwanensis* STM6044	90.7	98.7
*C. basilensis* MAG_BO332	78.2	97.4	24. *Cupriavidus ulmosensis*	CV2^T^	
*C. basilensis* SRS	76.9	97.2	*Cupriavidus* sp. CV2	100	100
*Cupriavidus* sp. UME77	73.3	96.7	*Cupriavidus* sp. CuC1	82.5	97.6
3. *Cupriavidus campinensis*	LMG 19282^T^		*Cupriavidus* sp. D39	78.8	96.8
*C. campinensis* LMG 19282^T^	100	100			
*C. campinensis* BWSM_125	98.56	99.8	25. *Cupriavidus agavae*	Only type strain sequenced	
*C. campinensis* G5	96.7	99.5	26.* “Cupriavidus gehlotii”*	Single strain	
*C. campinensis* CM30331	100	99.9	27. *Cupriavidus laharis*	Single strain	
*C. campinensis* MJ1	72.9	96.9	28. *Cupriavidus yeoncheonensis*	Single strain	
*C. campinensis* S14E4C	97.6	99.6			
*Cupriavidus* sp. MCTROC05	97.1	99.6	Novel *Cupriavidus* species	dDDH	ANI
*Cupriavidus* sp. nbed5b37 (1)	97.5	99.5	*Cupriavidus* sp. nov. 1	ip230pp230	
*Cupriavidus* sp. SIMBA	97.5	99.4	*C. taiwanensis* ip230pp230	100	100
4. *Cupriavidus cauae*	MKL01^T^		*C. taiwanensis* JHI1662	99.9	99.9
*C. cauae* MKL01^T^	100	100	*C. taiwanensis* LMG 19425	95.7	99.2
*C. cauae* PHS1	86	98.7	*C. taiwanensis* STM3511	97.2	99.5
*C. gilardii* W22	87.8	98.8	*C. taiwanensis* STM3679	97	99.5
*Cupriavidus* sp. DB3	86.1	98.7	*C. taiwanensis* STM3681	97.2	99.5
*Cupriavidus consociatus*	LEh25^T^		*C. taiwanensis* STM3711	97.1	99.5
*C. consociatus* LEh25^T^	100	100	*C. taiwanensis* STM6150	100	99.9
*C. consociatus* LEh21	99.9	99.9	*C. taiwanensis* STM8560	97.1	99.5
5. *Cuprividus gilardii*	ATCC 700815^T^		*C. taiwanensis* STM8561	97.1	99.5
*C. gilardii* ATCC 700815^T^	100	100	*C. taiwanensis* STM8564	97.1	99.5
*C. gilardii* CCUG 38401^T^	100	99.9	*C. taiwanensis* STM8565	97.1	99.5
*C. gilardii* CR3	99.1	99.7	*C. taiwanensis* TPIG6a	97.2	99.5
*C. gilardii* CY1	87.7	98.5	*C. taiwanensis* TPUD276	97.1	99.5
*C. gilardii* CY2	89.7	98.8	*Cupriavidus* sp. nov. 2	MAPUD101	
*C. gilardii* CY3	87.5	98.6	*C. taiwanensis* MAPUD101	100	100
*C. gilardii* CY4-1	98.3	99.6	*C. taiwanensis* mpp11	88.5	98.5
*C. gilardii* CY4-2	98.3	99.6	*C. taiwanensis* mpp13	89	98.6
*C. gilardii* FDAARGOS639	87.1	98.5	*C. taiwanensis* STM6116	91.2	98.9
*C. gilardii* JCM 11283	99.9	99.9	*C. taiwanensis* STM6117	91.2	98.9
*C. gilardii* M5	99.1	99.7	*C. taiwanensis* STM6119	91.2	98.9
*C. gilardii* QJ1	89.2	98.7	*C. taiwanensis* STM8556	89	98.6
*C. gilardii* WM02	85.8	98.4	*C. taiwanensis* STM8557	89	98.6
*Cupriavidus* sp. ISTL7	92.1	98.8	*C. taiwanensis* STM8558	89	98.6
7. *Cupriavidus lacunae*	S23^T^		*Cupriavidus* sp. nov. 3	EM10	
*C. lacunae* S23^T^	100	100	*Cupriavidus* sp. EM10	100	100
*C. necator* NH9	72.4	96.3	*Cupriavidus* sp. M04BS1SP1A44MG	99.3	99.9
8.* “Cupriavidus malaysiensis”*	USMAA1020^T^		*Cupriavidus* sp. M18BS1SP1A44MG	98.2	99.6
"*C. malaysiensis*" USMAA1020^T^	100	100	*Cupriavidus* sp. M27BS1SP1A44MG	99.6	99.9
*Cupriavidus* sp. USMAA24	93.3	99.2	*Cupriavidus* sp. M54BS1SP1A44MG	99.6	99.9
*Cupriavidus* sp. USMAHM13	93.9	99.3	*Cupriavidus* sp. M55BS1SP1A44MG	99.7	99.8
9. *Cupriavidus metallidurans*	CH34^T^		*Cupriavidus* sp. nov. 4	ALE26	
*C. metallidurans* CH34^T^	100	100	*C. necator* ALE26	100	100
*C. metallidurans* 162430002–4	91.8	99	*C. necator* FDAARGOS1030	99.8	99.9
*C. metallidurans* BS1	79.2	97.4	*C. necator* H16Nottingham	99.8	99.9
*C. metallidurans* FDAARGOS_675	100	99.99	*C. necator* H16Goettingen	99.8	99.9
*C. metallidurans* H1130	78.2	97.3	*C. necator* NBRC102504	100	99.9
*C. metallidurans* MAG71	92.3	99	*C. necator* PHE36a	95.4	99.3
*C. metallidurans* NA1	99.8	99.9	*Cupriavidus* sp. GA33	95.8	99.4
*C. metallidurans* NA4	92.8	99.1	*Cupriavidus* sp. nov. 5	STM6132	
*C. metallidurans* NBRC101272	88.2	98.6	*C. taiwanensis* STM6132	100	100
*C. metallidurans* NDB2Meth2	88.7	98.6	*C. taiwanensis* STM6162	100	99.9
*C. metallidurans* NDB3NO24	88.6	98.6	*C. taiwanensis* SWF65033	79.6	97.5
*C. metallidurans* NDB4MOL1	72.3	96.5	*C. taiwanensis* SWF66322	95.1	99.2
*C. metallidurans* NE12	72.4	96.6	*C. taiwanensis* SWF66324	82.6	97.9
*C. metallidurans* Ni2	89.9	98.7	*C. taiwanensis* QL117	100	100
*C. metallidurans* YL2	79.9	97.5	*Cupriavidus* sp. SS3	94.7	98.8
*C. metallidurans* ZM02	93.3	99	*Cupriavidus* sp. nov. 6	27098_8_120	
*C. metallidurans* ZM16	93.3	99	*C. gilardii* 27098_8_120	100	100
*Cupriavidus* sp. HMR1	72.2	96.5	*C. gilardii* BF20-02S	99.4	99.8
*Cupriavidus* sp. JdFR90	93.6	98.8	*C. gilardii* USM5	90.3	98.8
*Cupriavidus* sp. SHE	76.7	97.2	*C. gilardii* VKM-3265D	99.4	99.7
*Cupriavidus* sp. TKC	72.3	96.4	*Cupriavidus* sp. nov. 7	2SB	
*Cupriavidus* sp. UBA2010	72	96.5	*Cupriavidus* sp. 2SB	100	100
*Cupriavidus* sp. UBA2027	72.4	96.5	*Cupriavidus* sp. DLD2	89.4	98.8
*Cupriavidus* sp. UBA2526	72.3	96.6	*Cupriavidus* sp. SWY13	87.3	98.6
*Cupriavidus* sp. UBA2534	72.2	96.6	*Cupriavidus* sp. nov. 8	OTU4054	
*Cupriavidus* sp. UBA8761	72.4	96	*Cupriavidus* sp. OTU4054	100	100
*Cupriavidus* sp. UBA8769	72.5	96	*Cupriavidus* sp. OTU4895	100	99.9
10.* “Cupriavidus mimosae”*	WGtm5^T^		Cpauculus FDAARGOS664	94.3	99.2
*C. mimosae* WGtm5^T^	100	100	*Cupriavidus* sp. nov. 9	LMG 19,464	
*C. taiwanensis* STM6083	92.9	99.1	*C. taiwanensis* LMG 19464	100	100
*C. taiwanensis* SWF66294	93	99	*C. taiwanensis* S2-1-W	90.2	98.8
11. *Cupriavidus nantongensis*	X1^T^		*C. taiwanensis* USM6	91	98.7
*C. nantongensis* X1^T^	100	100	*Cupriavidus* sp. nov. 10	Agwp2	
*C. nantongensis* E324	81.2	97.7	*Cupriavidus* sp. Agwp2	100	100
12. *Cupriavidus necator*	N-1^T^		*Cupriavidus* sp. P10	92.6	98.9
*C. necator* N1^T^	100	100	*Cupriavidus* sp. TA19	96.9	99.5
*C. necator* ATCC 43291^T^	99.62	99.9	*Cupriavidus* sp. nov. 11	JZ4	
*C. necator* C39	71.5	96	*C. gilardii* JZ4	100	100
*C. necator* CR12	75.9	96.1	*Cupriavidus* sp. HPCL	87.3	98.5
*C. necator* H850	94.9	98.9	*Cupriavidus* sp. UGS1	87.5	98.6
*Cupriavidus* sp. BIC8F	69.6	95.9	*Cupriavidus* sp. nov. 12	X32	
*Cupriavidus* sp. KK10	94	98.8	*C. oxalaticus* X32	100	100
*Cupriavidus* sp. MCTROC19	75.2	96.7	*Cupriavidus* sp. IKTO18	94.4	99.2
*Cupriavidus* sp. SK4	72.8	95.8	*Cupriavidus* sp. L7L	87.9	98.5
13.* “Cupriavidus neocaledonicus”*	STM 6070^T^		*Cupriavidus* sp. nov. 13	GW210006_S54	
*C. neocaledonicus* STM6070^T^	100	100	*C. basilensis* GW210006_S54	100	100
*C. neocaledonicus* STM6082	99.9	99.9	*Cupriavidus* sp. SK3	91.4	98.6
*C. neocaledonicus* STM6160	99.9	99.9	*Cupriavidus* sp. nov. 14	KF708	
14. *Cupriavidus numazuensis*	LMG 26411^T^		*C. basilensis* KF708	100	100
*C. numazuensis* LMG 26411^T^	100	100	*Cupriavidus* sp. WS	86.7	98.5
*C. numazuensis* JCM 9086	100	99.9	*Cupriavidus* sp. nov. 15	Bin315	
15. *Cupriavidus oxalaticus*	Ox1^T^		*Cupriavidus* sp. Bin315	100	100
*C. oxalaticus* Ox1^T^	100	100	*Cupriavidus* sp. Bin721	95.8	98.9
*C. oxalaticus* HAMBI2164	99.9	99.9	*Cupriavidus* sp. nov. 16	CER94	
*C. oxalaticus* LMG 2235^T^	100	99.9	*Cupriavidus* sp. CER94	100	100
*C. oxalaticus* NBRC13593^T^	99.9	99.9	*Cupriavidus* sp. U2	79.2	97.2
*C. oxalaticus* Ox1mCherry	99.9	99.9	*Cupriavidus* sp. nov. 17	FDAARGOS614	
*C. oxalaticus* T2	90.4	98.9	*C. pauculus* FDAARGOS614	100	100
16. *Cupriavidus pampae*	LMG 32289^T^		*Cupriavidus* sp. SZYC1	89.7	98.7
*C. pampae* LMG 32289^T^	100	100	*Cupriavidus* sp. nov. 18	UYMSc13B	
*C. pampae* CCUG45425	100	99.8	*Cupriavidus* sp. UYMSc13B	100	100
17. *Cupriavidus pauculus*	CCUG 12507^T^		*C. necator* UYPR2512	70.4	95.4
*C. pauculus* CCUG 12507^T^	100	100	Single *Cupriavidus* sp. strains belonging to novel species		
*C. pauculus* BHJ32i	90.6	98.7	*C. basilensis* 4G11		
*C. pauculus* ERR7163067_concoct_124	91	98.8	*C. basilensis* GW210010_S58		
*C. pauculus* FDAARGOS1472	100	99.9	*C. basilensis* OR16		
*C. pauculus* HI2903	90.5	98.7	*C. basilensis* UME74		
*C. pauculus* JCM11286	100	99.9	*C. gilardii* CSURQ4897		
*C. pauculus* KF709	88	98.5	*C. gilardii* J11		
*C. pauculus* MF1	89.5	98.6	*C. nantongensis* HB4B5		
*C. pauculus* MSL348	96.4	99.3	*C. necator* A51		
18. *Cupriavidus phytohabitans*	AcVe19-1a^T^		*C. necator* B9		
*C. phytohabitans* AcVe19-1a^T^	100	100	*C. necator* DS4286		
*C. phytohabitans* AcVe19-6a	99	99	*C. necator* EM36		
*Cupriavidus* sp. AMP6	98.9	98.9	*C. necator* QL140		
19. *Cupriavidus phytorum*	MLR2-44^T^		*C. necator* SHC23		
*C. phytorum* MLR244^T^	100	100	*C. oxalaticus* JPY540		
*C. phytorum* LMG 19430	99.9	99.9	*C. pauculus* 19C6		
20. *Cupriavidus pinatubonensis*	LMG 23994^T^		*C. pauculus* UM1		
*C. pinatubonensis* LMG 23994^T^	100	100	*C. taiwanensis* STM3521		
*C. pinatubonensis* CGMCC112285	94	99.2	*Cupriavidus* sp. 731DF5525		
*C. pinatubonensis* HN2	76.6	97	*Cupriavidus* sp. AU9028		
*C. pinatubonensis* SM52	74.6	96.7	*Cupriavidus* sp. BIS7		
*C. necator* 5	76.5	97	*Cupriavidus* sp. CPBEBSR25		
21. *Cupriavidus plantarum*	ASC-64^T^		*Cupriavidus* sp. D384		
*C. plantarum* ASC-64^T^	100	100	*Cupriavidus* sp. IDO		
*C. plantarum* EM05	92.1	99	*Cupriavidus* sp. NPDC089707		
*C. plantarum* EM19	92.1	99	*Cupriavidus* sp. OV038		
*C. plantarum* EM20	92	99	*Cupriavidus* sp. OV096		
*C. plantarum* LMG 26,296	100	99.9	*Cupriavidus* sp. SMAGNO102		
*C. plantarum* MA1-2za	92.5	99.1	*Cupriavidus* sp. SRR17738104		
*C. plantarum* MA1-4a	92.6	99.1	*Cupriavidus* sp. WKF15		
*C. plantarum* MA2-19b	92.1	99	*Cupriavidus* sp. YR651		
*C. plantarum* SLV-132	93.8	99			

dDDH, digital DNA-DNA performed with the Genome-to-Genome Distance Calculator (GGDC, http://ggdc.dsmz.de/ggdc.php·9). ANI, average nucleotide identity using fast ANI with the program anvi’o^41^. Species names with quotation marks mean names not validated under the International Code of Nomenclature of Prokaryotes^30^.

(1) Contaminated genome.

Thus, based on our results, *C. alkaliphilus* comprises nine strains, including *C. taiwanensis* cmp52 (71.52% dDDH), which was reassigned to this species. Chen et al.^23^ included 16 strains within *C. alkaliphilus*. However, seven of these strains have ANI values around 95%, but their dDDH values range from 65.2 to 65.7% to the type strain ASC-732^T^, which is below the established cut-off (70%). It is also important to note that Chen et al.^23^ used strain SrBR-232 for the genomic comparison. The authors also indicated that strain *C. alkaliphilus* MLR2-44 is mislabeled; however, this strain, along with *C. taiwanensis* LMG 19430, had already been described as *Cupriavidus phytorum* in early 2025^5^. “*C. ariensis*”, a new, unpublished genomic species (Raúl Platero, personal communication), comprises two strains. *C. basilensis* was split into several genomic species, keeping the type strain and three more strains (one unclassified). Chen et al.^23^ included *C. basilensis* 4G11, but the ANI is 95% and dDDH 62.5% to the type strain; thus, it does not correspond to this species. They missed adding *Cupriavidus* sp. MAG_BO332, SRS, and UME77. *C. campinensis* comprises nine strains, three of which are unclassified, and one of the last is an uncultured *Cupriavidus* with a genome marked as contaminated in the NCBI. *C. cauae* is formed by four strains: two already named *C. cauae*, one named *C. gilardii*, and an unclassified strain. *C. consociatus* comprises two strains. *C. gilardii* was divided into a few groups, one of which contained the type strain and 12 additional strains, including one unclassified strain. Chen et al.^23^ included only five *C. gilardii* strains, whereas our analysis identified 13 strains. *C. lacunae* is formed by the type strain and a *C. necator* strain. “*C. malaysiensis"* was described with a single strain, which matched two other unclassified *Cupriavidus* strains. *C. metallidurans* was split into several genomic species; the one containing the type strain comprises 27 strains, among them 10 unclassified strains. Chen et al.^23^ report only 14 strains and used a reference strain rather than the type strain CH34^T^. A recent proposal (in a preprint) is “*C. mimosae”* comprised of the type strain and two *C. taiwanensis* strains. *C. nantongensis* is formed of two strains. *C. necator* contains eight strains, with four of them unclassified and the type strain sequenced twice. Chen et al.^23^ also reported eight strains; however, the dDDH of strains FDAARGOS1030 (68.3%), H16 (68.3%), NBRC 102,504 (68.9%), and PHE3-6 (68.6%) are below the cut-off value. The authors missed including strains BIC8F, KK10, MCTROC19, and SK4. “*C. neocaledonicus*” is formed by three strains, and *C. numazuensis* by two strains*. C. oxalaticus* comprises four strains, with the type strain sequenced three times (Ox1^T^, LMG 2235^T^, and NBRC 13593^T^). Chen et al.^23^ included five strains, of which three are the sequenced type strains. *C. pampae* contains two strains, and *C. pauculus* nine strains. Chen et al.^23^ reported five *C. pauculus* strains compared with a reference strain (FDAARGOS1472) rather than the type strain (CCUG 12507^T^). *C. phytohabitans* comprises three strains (one assigned with polyphasic taxonomy in this study), and *C. phytorum* comprises two strains. Chen et al.^23^ reported this species as group 18. *C. pinatubonensis* is composed of five strains, one of which was previously named *C. necator*. *C. plantarum* comprises nine strains, and *C. respiraculi* comprises three strains, with the type strain sequenced twice. *C. taiwanensis* was split into several species, but the one containing the type strain comprises 10 strains. Chen et al.^23^ reported nine strains, missing *C. taiwanensis* SMAGNO117. Moreover, the authors noted that, because *C. taiwanensis* is a species complex that includes several new species, it is no longer reasonable to use LMG 19424^T^ as the type strain. However, this goes against the International Code of Nomenclature of Prokaryotes^29^; the only reason to change a type strain is when the original is lost, then a neotype strain is proposed. When a species is divided into multiple species, the species name is retained for the species that includes the type strain; thus, a new type strain must then be designated for the new species. The recently described *C. ulmosensis* is formed of three strains.

Moreover, the species described with a single strain, or with only the genome of one sequenced strain, are *C. agavae*, “*C. gehlotii*”, *C. laharis,* and *C. yeoncheonensis*. The remaining *Cupriavidus* strains form 18 novel genomic species, each containing 2 to 14 strains. Furthermore, 30 single strains correspond to novel genomic species.

Chen et al.^23^ reported 11 new species, instead of the 18 proposed in our study. This is because the authors analyzed only 97 strains, and our study comprises more than 250 strains.

A phylogenomic analysis of all *Cupriavidus* strain genomes was performed using PhyloPhlAn (Fig. 5). Strain names remained unchanged and corresponded to those in the NCBI database. There were a few inconsistencies in the phylogenomic tree and the groups formed in Table 3, which were based on dDDH and ANI results. Those were *C. necator*, *C. campinensis*, *C. basilensis,* and *Cupriavidus* sp. nov. 18. These species contained unrelated strains (with low dDDH and ANI values). The results presented in this analysis indicate that more *Cupriavidus* strains remain to be described by proper polyphasic taxonomy.

## Conclusion

Given the results of modern taxonomy, which combines classical phenotyping, chemotaxonomy, and genotyping analysis, presented in this study, the strains AcVe19-1a^T^, AcVe19-1b, AcVe19-6a, and AMP6 represent a novel species proposed as *Cupriavidus phytohabitans* sp. nov. Additionally, comparative genomic analysis reveals that the genus *Cupriavidus* comprises more species than those currently described.

### Description of *Cupriavidus phytohabitans* sp. nov.

*Cupriavidus phytohabitans* (phy.to.ha’bi.tans. Gr. neut. n. *phyton*, plant; L. pres. part. *habitans*, inhabiting; N.L. part. adj. *phytohabitans*, plant-inhabiting, referring to *Phaseolus vulgaris* root nodules, where the type strain was isolated).

Cells stain Gram-negative, and rods are 1.3 μm long and 0.5 μm wide. Colonies were circular, convex, glossy, and beige-colored in the LB medium. The strains grew on LB at 20, 25, 30, 37, and YM, and on MacConkey agar at 30, 37, with growth at 42 °C being strain-dependent. They grew on LB up to 1% NaCl, but AcVe19-1a^T^ up to 2% NaCl. The pH range for growth was 6.0 – 9.0, but AcVe19-1a^T^ grew from 5.0 to 9.0. The alkalinization of L-lactate and succinate was positive. The activity of gamma-glutamyl-transferase, L-proline-arylamidase, tyrosine arylamidase, and phosphatase was positive. The strains assimilate citrate, malonate, L-malate, Ellman, and L-lactate. Total polar lipids consisted of phosphatidylethanolamine (PE), diphosphatidylglycerol (DPG), phosphatidylglycerol (PG), cardiolipin (CL), and unknown amino lipids. The genome of the novel species harbors nitrogen-fixing and nodulation genes, enabling it to nodulate *P. vulgaris* and *Mimosa* plants. However, nitrogen is unable to be fixed in either the free-living or the nodule.

Type strain AcVe19-1a^T^ (= TSD-313^T^ = CDBB B-2084^T^) was isolated from root nodules of *P. vulgaris* used as a trap plant inoculated with rhizospheric soil from *Acacia* sp. collected in Veracruz, Mexico. The draft genome is 7.42 Mbp and has a G + C content of 65.7 mol%. The GenBank accession number of the 16S rRNA is MN830146, and the genome assembly accession number is GCA_017814995.1.

### Experimental procedures

#### Bacterial isolation

*Cupriavidus* strains AcVe19-1a^T^, AcVe19-6a, and AcVe19-6b were previously isolated from *P. vulgaris* root nodules^8^. Briefly, the experiment was carried out by mixing soil with vermiculite (3:1) in 325 mL pots. The soil was collected from the rhizosphere of *Acacia* sp. growing in the wild in Veracruz, Mexico (18°29′39.516’' N, 95°2′35.015’' W). *P. vulgaris* var. Negro Jamapa seeds were disinfected with 3% NaOCl for 10 min. The seeds were placed on 15% agar-water plates and incubated at 30 °C for 72 h. The germinated seeds were planted in 5 pots (one seed per pot) and maintained for 45 days in a greenhouse at 30 °C, with a 14 h light and 10 h dark cycle. The bacterial isolation was achieved by randomly selecting five legume nodules from each plant and washing them three times with sterile water. Then, the nodules were disinfected as described above. The final washing water was placed in LB medium to verify surface disinfection. The nodules were crushed with a plastic pestle in 40 mL of sterile water. The nodule suspension was inoculated onto plates with yeast extract mannitol (YM) medium containing 5 g of mannitol. The plates were incubated at 30 °C for 3–5 days. After re-streaking for pure colonies, they were stored in 35% glycerol at -70 °C for later analysis.

### Phylogenetic analysis by 16S rRNA gene sequence

The 16S rRNA gene sequences from strains AcVe19-1a^T^ (MN830146), AcVe19-6a (MN830148), and AcVe19-6b (MN830149) were obtained earlier^8^. The 16S rRNA gene sequence from *Cupriavidus* sp. AMP6 is available with the accession number DQ530646. The 16S rRNA gene sequences from all type strains of *Cupriavidus* species were obtained to perform a phylogenetic analysis. To achieve the latter, an alignment was performed using MUSCLE^30^. Then, using the maximum likelihood method and the GTR + G + I model in the software PhyML 3.1, a phylogenetic tree was constructed and displayed using MEGA version 11^31^. Bootstrap analysis was performed with 1000 replications.

### Whole genome sequencing and comparative analysis

The strains AcVe19-1a^T^ and AcVe19-6a were grown in 40 mL LB and incubated overnight at 30 °C. The total DNA was isolated using the method of Moore and Dowhan^32^. The genome sequence was obtained at Novogene (https://en.novogene.com/) using the Illumina Platform PE150 with libraries paired-sequenced (2 × 350 bp). The quality of raw sequencing data was evaluated using FastQC v0.11.9^33^. Adapter screening and quality filtering of reads were performed with Trimmomatic 0.39^34^. De novo genome assemblies were performed with SPAdes 3.14 program^35^. Metrics such as N50 and misassembles were obtained with QUAST v5.0.2^36^. Annotation was performed using the NCBI Prokaryotic Genome Annotation Pipeline at the National Center for Biotechnology Information (NCBI). The dDNA-DNA values were estimated using formula 2 of the Genome-to-Genome Distance Calculator (GGDC, http://ggdc.dsmz.de/ggdc.php#9), with 70% as the level of similarity used for species delineation^37^. The ANI values^38^ were calculated using fastANI, a program within anvi’o^39^. A phylogenomic analysis was inferred using the program PhyloPhlAn 3.0, which analyses 400 universal markers using the maximum likelihood method^40^. The amino acid substitution model and bootstrap analysis (1000 replications) were performed using IQ-TREE^41^. The tree was displayed with iTOL v. 7 (https://itol.embl.de/). The study included all *Cupriavidus* strains.

### Morphology, physiology, and biochemical analysis

Strains AcVe19-1a^T^, AcVe19-6a, and AcVe19-6b were characterized following different phenotypic features. Colony morphology was determined on LB agar plates after two days at 30 °C. Temperature-dependent growth was evaluated on LB at 20, 25, 30, 37, and 42 °C and on YM, and MacConkey agar plates at 30, 37, and 42 °C after two days at 30 °C. Salt tolerance in modified LB (without NaCl) agar plates was examined by adding 0.5, 1, 2, 3, 4, and 5% NaCl, incubating two days at 30 °C. The effect of pH on bacterial growth was established in liquid LB adjusted with the following 1X buffers: a glycine–HCl buffer for ranges of pH 1.0 – 3.0; an acetate-based buffer for pH 4.0 – 5.0; a citric acid-phosphate buffer for pH 6.0 – 7.0; a Tris–HCl buffer for pH 8.0 – 9.0; a glycine–NaOH buffer for pH 10.0 – 12.0; and a KCl-NaOH buffer for pH 13.0^42^. The tubes were incubated for five days at 30 °C (150 rpm). Biochemical tests were performed using the VITEK 2 System with the VITEK 2 GN card, following the manufacturer’s instructions (BIOMÉRIUX). Cells were observed using scanning electron microscopy (SEM), with the cells grown in BSE^11^. The cells were fixed in a solution containing 2.5% glutaraldehyde in 0.1 M cacodylate buffer (pH 7.2) and post-fixed for 1 h with 1% osmium tetroxide (OsO_4_) in 0.1 M cacodylate buffer, followed by dehydration with increasing concentrations of ethanol (60, 70, 80, 90, and 100%). The samples were subjected to critical point and gold-coated. Coverslips containing the samples were attached to aluminum holders and analyzed using a scanning electron microscope JEOL model JSM 5800-LV.

### Chemotaxonomic analysis

Whole-cell proteins were analyzed as described previously by using SDS-PAGE^43^ for AcVe19-1a^T^, AcVe19-6a, AcVe19-6b, *C. consociatus* LEh25^T^, *C. oxalaticus* NBRC 13593^T^, *C. necator* N-1^T^, and *C. taiwanensis* LMG 19424^T^. Briefly, bacteria were grown in Jain and Patriquin^44^ medium with reciprocal shaking (200 rpm), for 15 h at 29 °C, and 1.0 mL samples were harvested by centrifugation at 12,300 × *g* for 10 min. The pellet was resuspended in 70 μL of 0.125 M Tris–HCl, 4% SDS, 20% glycerol, and 10% mercaptoethanol at pH 6.8. Aliquots of 10 μL were used for SDS-PAGE. Polar lipids were analyzed for strains AcVe19-1a^T^, *C. consociatus* LEh25^T^, and *C. oxalaticus* Ox1^T^. To this purpose, strains were grown in LB liquid media for 16 h at 29 °C, and the polar lipids were extracted following the Bligh and Dyer^45^ technique. The chloroform phase was used for lipid analysis on TLC plates by two-dimensional separation^46^. Total polar lipids were observed by spraying with ANS reagent (8-anilino-1-naphtalenesulfonic acid)^47^ and iodine vapor^48^. Polar lipids were identified by their migration patterns, combined with specific staining. Lipids containing amino groups and glycolipids were determined using the ninhydrin and periodate-Schiff techniques, respectively, as described previously^49,50^.

### Phylogenetic analysis of ubiquinones

The in silico analysis of ubiquinones was performed by extracting the amino acid sequences corresponding to UbiA, UbiB, UbiD, UbiE, UbiF, UbiG, UbiH, UbiJ, and UbiX from the genome sequences of all type strains of *Cupriavidus* species. The sequences for each protein were aligned individually with Clustal Omega (https://www.ebi.ac.uk/Tools/msa/clustalo/) and then concatenated employing the software Mesquite v3.81 (http://www.mesquiteproject.org/?HistoryPanel=open). The phylogenetic analysis was performed using the maximum likelihood method and the BLOSUM62 model. The tree was displayed with MEGA version 11^31^.

### Resistome analysis

The prediction of the antibiotic resistome was analyzed using CARD RGI (Comprehensive Antibiotic Resistance Database, Resistance Gene Identifier) software^51^.

### Nodulation test

*Mimosa pudica* seeds were treated with H_2_SO_4_ for 5 min, then washed five times with sterile water. Next, *M. pudica,* and *P. vulgaris* var. Negro Jamapa seeds were disinfected with 10% sodium hypochlorite for 10 min and washed five times with sterile water. The seeds were placed on 15% agar-water plates and incubated at 30 °C for 72 h in the dark. The germinated seeds were sown in sterile vermiculite in 325 mL pots and inoculated with 2 mL of a bacterial suspension containing approximately 1 × 10^8^ cells per milliliter. The pots were incubated for 45 days in a greenhouse at 30 °C with a 14 h light and 10 h dark cycle, and watered with Fahraeus solution^52^. The experiment was conducted with three plants per treatment in individual pots, and the experiment was repeated once more with another three plants per treatment. The treatments were: a) control inoculated with water; b) inoculation with strains AcVe19-1a^T^, and AcVe19-6a; and c) inoculation with two *Rhizobium* sp. strains: MpTa5-8a (16S rRNA gene accession number MN830136)*,* and AcVe20-20b (MN830153), as positive controls. After the incubation period, the re-isolation of the inoculated strains was performed by pooling up to ten nodules from each plant in each treatment. The strains were identified by 16S rRNA gene sequence analysis, as described above.

Before bacterial isolation from nodules, the roots with the nodules from all treatments were gently washed with sterile water and placed in 100 mL vials. The vials were sealed with rubber seals, and 5% of the total air volume was extracted and replaced with acetylene^11^. The roots were left at room temperature for 8 h. Next, nitrogen fixation was measured indirectly by the reduction of acetylene to ethylene, with a Clarus 580 gas chromatograph (PerkinElmer). Additionally, the strains were individually tested for nitrogen fixation by growing them in 10 mL vials containing 5 mL of semisolid (2.3 g/L) YM medium, lacking nitrogen, for 3 days. Then, the cotton plug was replaced with a rubber seal, and 10% of the air was replaced with acetylene. The vials were incubated overnight at 30 °C. The next day, the nitrogen fixation was measured, as mentioned before.

### Genetic organization of nitrogen fixation and nodulation genes

The genome sequences of strains AcVe19-1a^T^ and AcVe19-6a were explored for the presence of nitrogen-fixation and nodulation genes. For nitrogen fixation, *nif, fix*, and *fdx* genes were screened in AcVe19-1a^T^, AcVe19-6a, AMP6, *C. taiwanensis* LMG 19424^T^*, C. consociatus* LEh25^T^, *Paraburkholderia phymatum* STM815^T^, and *Bradyrhizobium* sp. DOA9. A phylogenetic analysis of NifH was performed using several nitrogen-fixing bacteria, mainly from the genera *Paraburkholderia*, *Cupriavidus*, and *Trinickia,* with *Rhizobium etli* CFN42^T^ and *Bradyrhizobium japonicum* ATCC 10324^T^ used as the outgroup. The phylogenetic analysis used the maximum likelihood method and the amino acid substitution model BLOSUM62. The tree was displayed with MEGA version 11^31^. Bootstrap analysis was performed with 1000 replications.

For the analysis of nodulation genes, the *nod*, *nol,* and *noe* genes were searched in the genomes mentioned for nitrogen fixation. A phylogenetic analysis of NodC was conducted using the novel species and other species from the genera *Cupriavidus*, *Paraburkholderia*, *Trinickia*, *Rhizobium,* and *Mesorhizobium*. The phylogenetic tree was performed similarly to the one for NifH.

## Supplementary Information

Supplementary Information 1.

Supplementary Information 2.

Supplementary Information 3.

Supplementary Information 4.

Supplementary Information 5.

Supplementary Information 6.

Supplementary Information 7.

Supplementary Information 8.

Supplementary Information 9.

## Data Availability

All the data have been deposited in the GenBank at the National Center for Biotechnology Information. The 16S rRNA genes were deposited under the accession numbers MN830146 (AcVe19-1a^T^), MN830148 (AcVe19-6a), and MN830149 (AcVe19-6b). The genome accession numbers for AcVe19-1a^T^, AcVe19-6a, and AMP6 are JAGIQC000000000, JAGIQB000000000, and AUFE00000000, respectively.
