# Supplementary material for: Phylogenomic analysis shows underestimated species within Cupriavidus and the new species Cupriavidus phytohabitans sp. nov
Source: Sci Rep. 2026 Feb 13;16:8774. doi: 10.1038/s41598-026-39004-6 (PMC12982536; doi:10.1038/s41598-026-39004-6)

Figure S1. Phylogenetic tree of *Cupriavidus* species based on the comparison of 16S rRNA gene sequence. The analysis used the maximum likelihood method and the GTR + G + I model. The numbers in the branches correspond to the bootstraps. In parentheses are the sequence accession numbers. In red is indicated the novel species. The bar shows the differences between sequences.

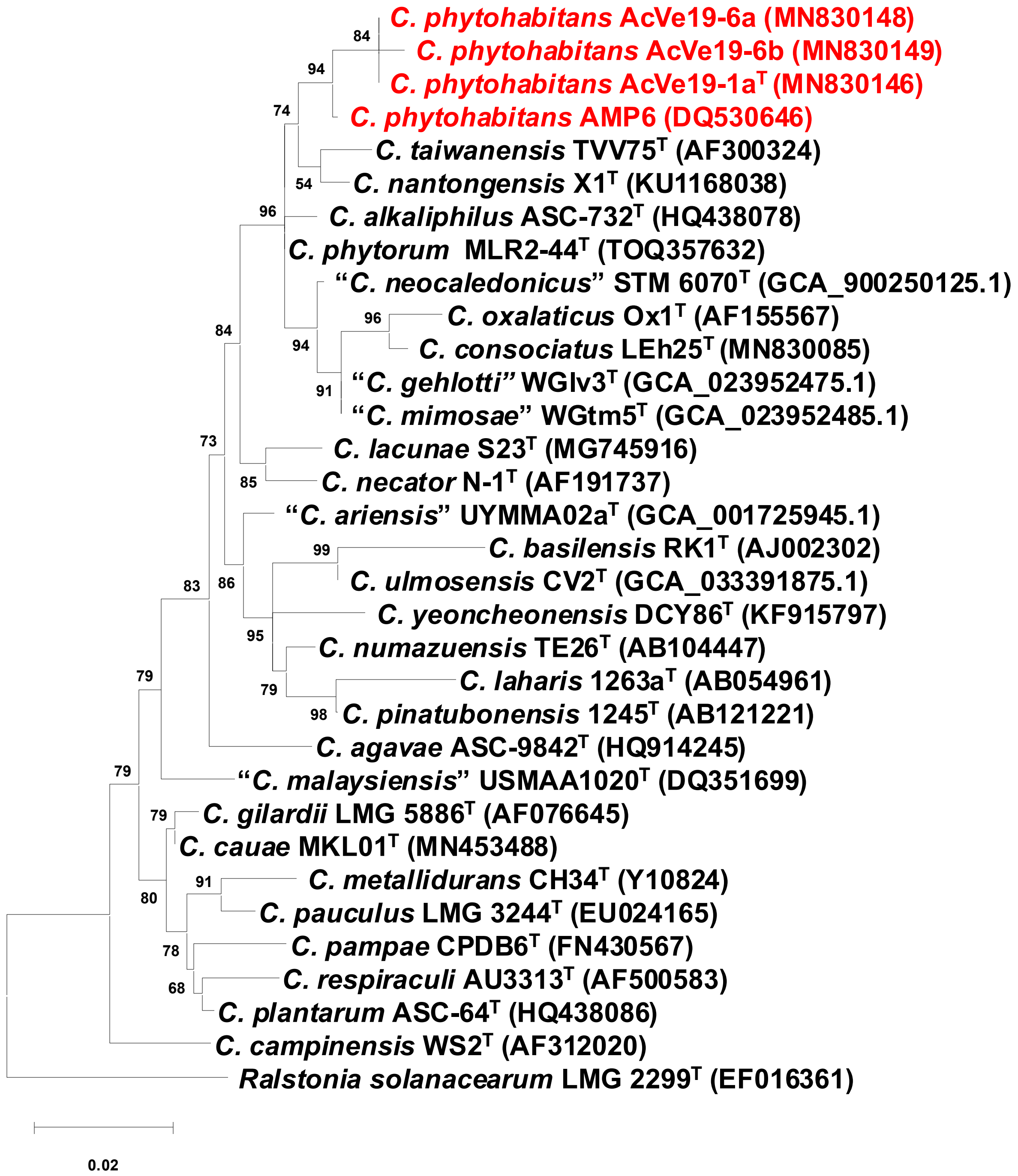

Supplement: Supplementary file 1 — Supplementary Information 1. [file 41598_2026_39004_MOESM1_ESM.pdf]
