# Supplementary figures and images for: Phylogenomic analysis shows underestimated species within Cupriavidus and the new species Cupriavidus phytohabitans sp. nov
Source: Sci Rep. 2026 Feb 13;16:8774. doi: 10.1038/s41598-026-39004-6 (PMC12982536; doi:10.1038/s41598-026-39004-6)

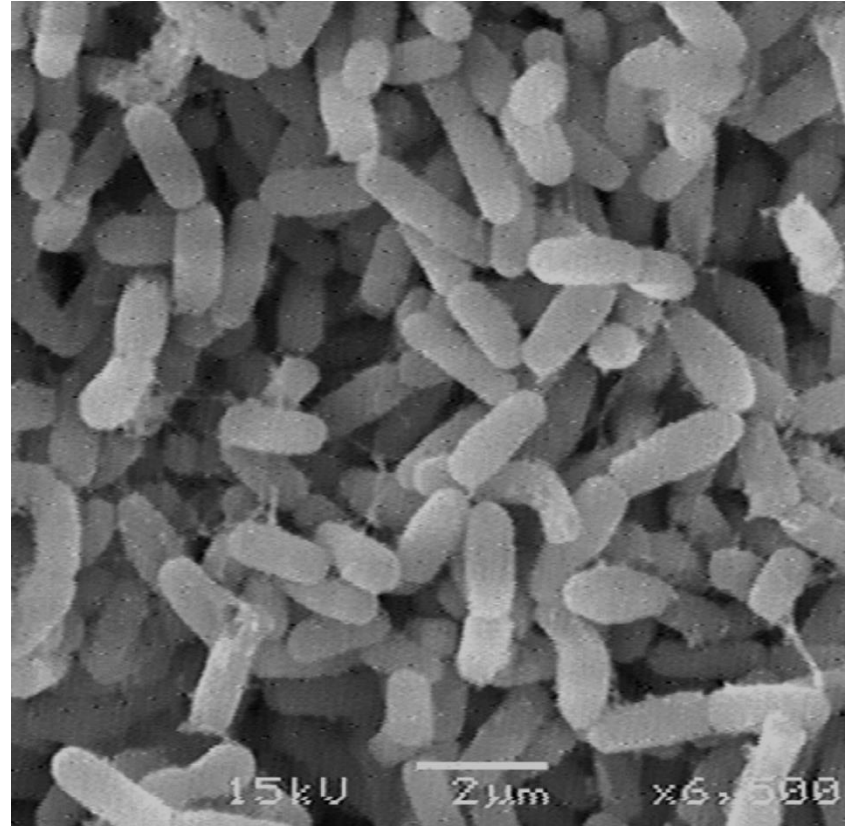

**Figure S2.** Scanning electron micrograph of *Cupriavidus phytohabitans* sp. nov. cells. Bar, 2 mm.

Supplement: Supplementary file 2 — Supplementary Information 2. [file 41598_2026_39004_MOESM2_ESM.pdf]
