# Supplementary material for: Phylogenomic analysis shows underestimated species within Cupriavidus and the new species Cupriavidus phytohabitans sp. nov
Source: Sci Rep. 2026 Feb 13;16:8774. doi: 10.1038/s41598-026-39004-6 (PMC12982536; doi:10.1038/s41598-026-39004-6)
Supplement: Supplementary file 3 — Supplementary Information 3. [file 41598_2026_39004_MOESM3_ESM.pdf]

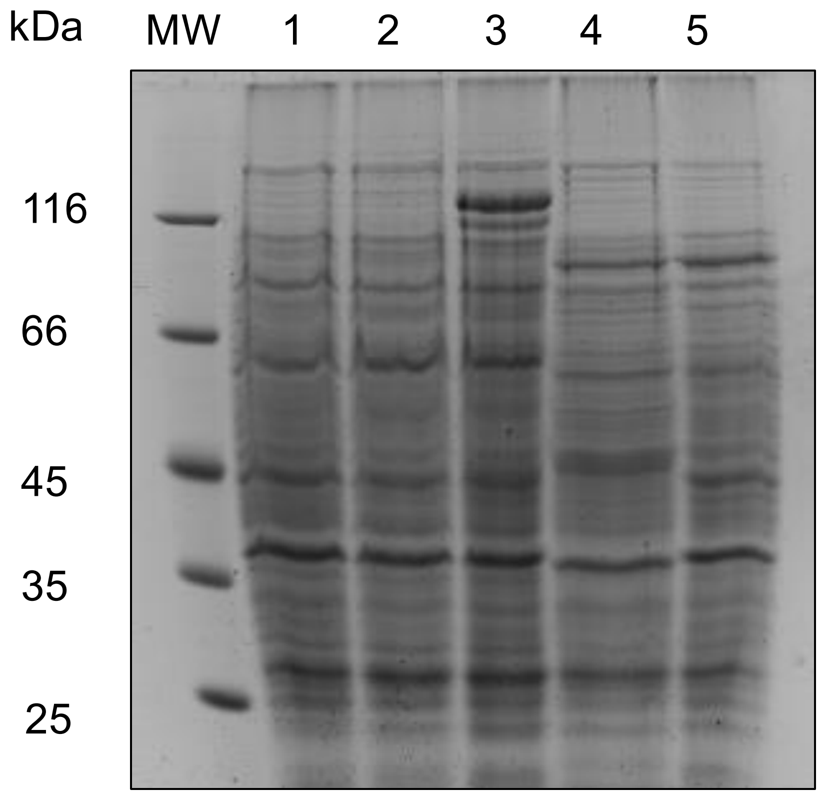


**Figure S3**. Protein electropherograms (SDS-PAGE) of *Cupriavidus phytohabitans* sp. nov. and close and relevant type strains of *Cupriavidus* species. Strains: 1, AcVe19-1a$^T$; 2, AcVe19-6a; 3, AcVe19-6b; 4, *C. oxalaticus* Ox1$^T$; 5, *C. consociatus* LEh25$^T$. MW, PageRuler molecular maker.
