# Supplementary material for: Phylogenomic analysis shows underestimated species within Cupriavidus and the new species Cupriavidus phytohabitans sp. nov
Source: Sci Rep. 2026 Feb 13;16:8774. doi: 10.1038/s41598-026-39004-6 (PMC12982536; doi:10.1038/s41598-026-39004-6)
Supplement: Supplementary file 4 — Supplementary Information 4. [file 41598_2026_39004_MOESM4_ESM.pdf]

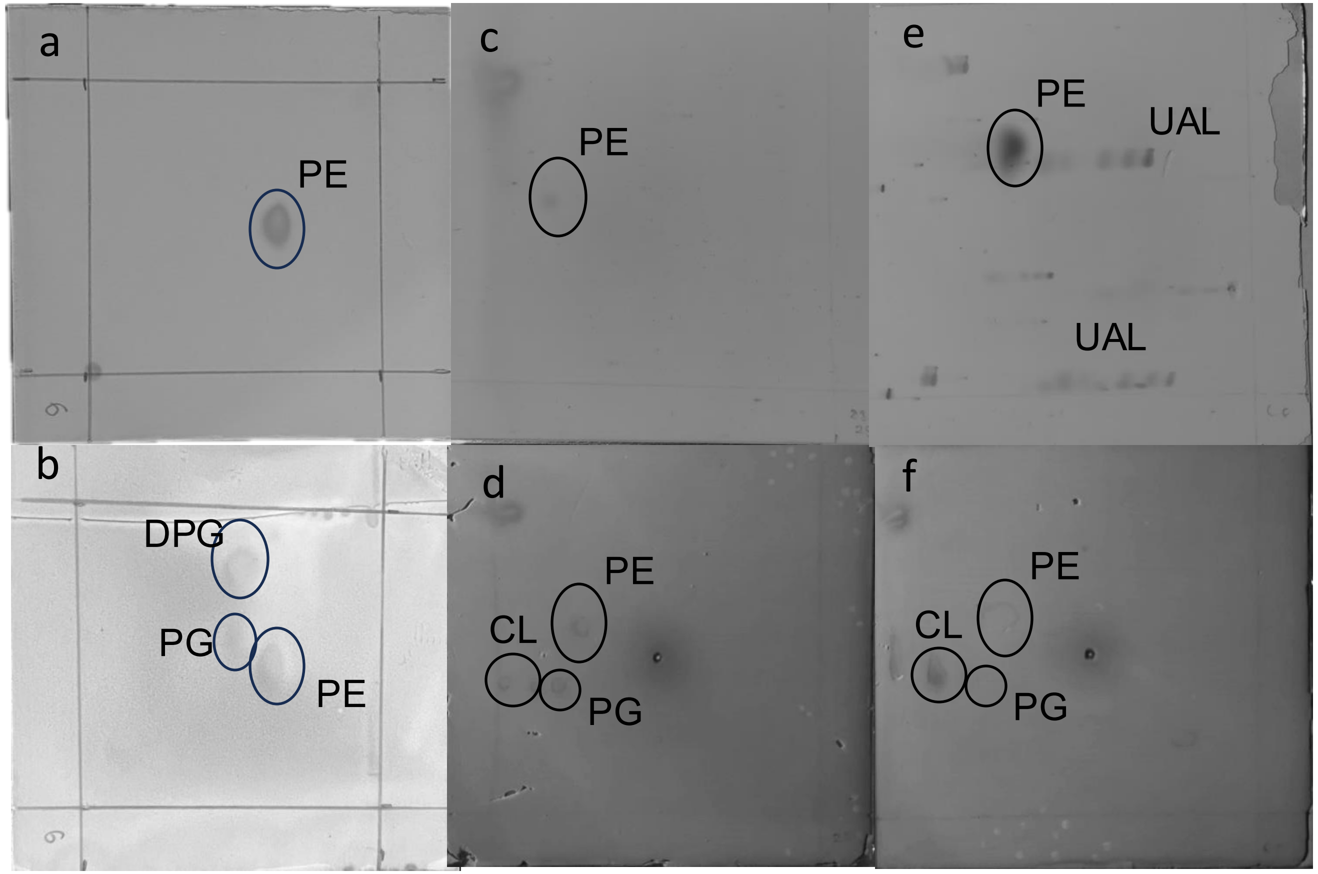

Figure S4. Membrane lipid profiles of *Cupriavidus phytohabitans* sp. nov. and related type strains of *Cupriavidus* species. a, b) *C. phytohabitans* sp. nov. AcVe19-1a^T. c, d) *Cupriavidus consociatus* LEh25^T. e, f) *Cupriavidus oxalaticus* Ox1^T. a, c, e) ninhydrin staining. b, d, f) Shiff's reagent staining. PE, phosphatidylethanolamine. DPG, diphosphatidylglycerol. PG, phosphatidylglycerol. UAL, unknown aminolipid. CL, cardiolipin.
