# Supplementary material for: Phylogenomic analysis shows underestimated species within Cupriavidus and the new species Cupriavidus phytohabitans sp. nov
Source: Sci Rep. 2026 Feb 13;16:8774. doi: 10.1038/s41598-026-39004-6 (PMC12982536; doi:10.1038/s41598-026-39004-6)
Supplement: Supplementary file 5 — Supplementary Information 5. [file 41598_2026_39004_MOESM5_ESM.pdf]

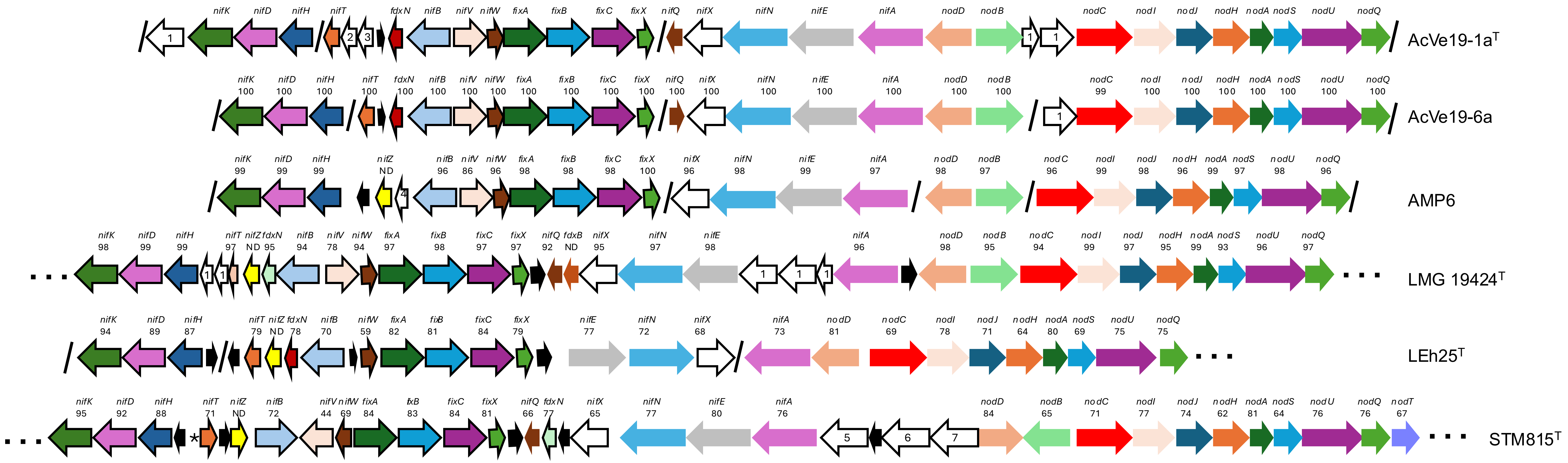

1. Transposase
2. Reverse transcriptase-like protein
3. Group II catalytic intron D1-D4-2
4. 4Fe-4S dicluster protein
5. DNA binding transcriptional LysR family regulator
6. Biotin carboxylase
7. ATP-grasp domain-containing protein

ND, not determined because strain AcVe19-1a^{T} does not contain that gene.
/ Different contigs
▪ ▪ ▪ Contig continues

Figure S5. Nodulation and nitrogen fixation gene organization in *Cupriavidus phytohabitans* sp. nov. and close and relevant symbiotic bacteria. Strains AcVe19-1a, AcVe19-6a, and AMP6 correspond to the novel species. Strain LMG 19424^{T} corresponds to *Cupriavidus taiwanensis*. Strain LEh25^{T} corresponds to *Cupriavidus consociatus.* Strain STM815^{T} corresponds to *Paraburkholderia phymatum*. The asterisk means other genes (homeodomain-containing protein, putative FmdB family regulatory protein, uncharacterized protein, hypothetical protein). The numbers below each gene correspond to the identity percentage with strain AcVe19-1a^{T}. The gene organization was obtained from the Integrated Microbial Genomes and Microbiomes at the Joint Genome Institute of the Department of Energy.
