# Supplementary material for: Phylogenomic analysis shows underestimated species within Cupriavidus and the new species Cupriavidus phytohabitans sp. nov
Source: Sci Rep. 2026 Feb 13;16:8774. doi: 10.1038/s41598-026-39004-6 (PMC12982536; doi:10.1038/s41598-026-39004-6)
Supplement: Supplementary file 7 — Supplementary Information 7. [file 41598_2026_39004_MOESM7_ESM.pdf]

Table S1. Genomic features of *Cupriavidus phytohabitans* sp. nov.

Genomic features	AcVe19-1a^T	AcVe19-6a
Genome length (bp)	7423665	7432279
Number of contigs	210	315
G + C content (%)	65.70	65.58
CDS	6923	6983
RNA genes	82	81
rRNA genes	9	10
5S rRNA	2	1
16S rRNA	3	3
23S rRNA	4	6
tRNA genes	69	67
Protein coding genes with function prediction	5653	5601
without function prediction	1270	1382
Protein coding genes with enzymes	1898	1856
Protein coding genes connected to KEGG pathways	2110	2083
not connected to KEGG pathways	4813	4900
Protein coding genes connected to KEGG Orthology (KO)	3490	3529
not connected to KO	3433	3529
Protein coding genes connected to MetaCyc pathways	1657	1619
not connected to MetaCyc pathways	5266	5364
Protein coding genes with COGs	5587	5526
with Pfam	5789	5723
with TIGRfam	1809	1775
with SMART	1337	1335
with SUPERFam	5746	5694
with CATH FunFam	4699	4630
in internal clusters	2591	2600
Protein coding genes coding signal peptides	688	688
Protein coding genes coding transmembrane proteins	1481	1476
COG clusters	2182	2179
Pfam clusters	2697	2723
TIGRfam clusters	1314	1308
Completeness (%)	97.92	97.86
Contamination (%)	0.58	2.35
