# Supplementary material for: Phylogenomic analysis shows underestimated species within Cupriavidus and the new species Cupriavidus phytohabitans sp. nov
Source: Sci Rep. 2026 Feb 13;16:8774. doi: 10.1038/s41598-026-39004-6 (PMC12982536; doi:10.1038/s41598-026-39004-6)
Supplement: Supplementary file 8 — Supplementary Information 8. [file 41598_2026_39004_MOESM8_ESM.pdf]

Table S2. Phenotypic features between *Cupriavidus phytohabitans* sp. nov. and the closest and relevant *Cupriavidus* species.

Phenotypic feature	***Cupriavidus phytohabitans* AcVe19-1a**	***Cupriavidus phytohabitans* AcVe19-6a**	***Cupriavidus phytohabitans* AcVe19-6b**	***Cupriavidus consociatus* LEh25^T**	***Cupriavidus oxalaticus* Ox1^T**	***Cupriavidus necator* N-1^T**	***Cupriavidus taiwanensis* LMG 19424^T**
Isolation source	*Phaseolus vulgaris* nodules	*Phaseolus vulgaris* nodules	*Phaseolus vulgaris nodules*	*Leucaena* sp. nodules	Alimentary tract earthworm	Soil	*Mimosa pudica* nodules
Location source	Veracruz, México	Veracruz, México	Veracruz, México	Chiapas, México	India	United States	Taiwan
Gram stain	–	–	–	–	–	–	–
Growth on LB agar at:							
20 °C	+	+	+	+	nd	nd	nd
25 °C	+	+	+	+	nd	nd	nd
30 °C	+	+	+	+	+	+	+
37 °C	+	+	+	+	+	+	+
42 °C	+	+	–	+	+	+	+
Growth on YM agar at:							
30 °C	+	+	+	+	+	+	+
37 °C	+	+	+	+	+	+	+
42 °C	+	–	–	+	+	+	+
Growth on MacConkey agar at:							
30 °C	+	+	+	+	+	+	+
37 °C	+	+	+	+	+	+	+
42 °C	+	–	+	+	+	+	+
Growth on LB agar + NaCl (%):							
0.0	+	+	+	+	+	+	+
0.5	+	+	+	+	+	+	+
1.0	+	+	+	+	+	+	+
2.0	+	–	–	+	+	+	+
3.0	–	–	–	–	–	–	+
4.0	–	–	–	–	–	–	–
5.0	–	–	–	–	–	–	–
Growth at pH values:							
1.0	–	–	–	–	–	–	–
2.0	–	–	–	–	–	–	–
3.0	–	–	–	–	–	–	–
4.0	–	–	–	–	–	–	–
5.0	+	–	–	–	–	–	–
6.0	+	+	+	+	–	+	+
7.0	+	+	+	+	–	+	+
8.0	+	+	+	+	+	+	+
9.0	+	+	+	+	+	+	+
10.0	–	–	–	–	–	–	–
11.0	–	–	–	–	–	–	–
12.0	–	–	–	–	–	–	–
13.0	–	–	–	–	–	–	–

H_2S production	–	–	–	–	–	–	–
Alkalinization of:							
L-lactate	+	+	+	+	+	+	+
Succinate	+	+	+	+	+	+	+
Activity of:							
Ala-Fe-Pro-arylamidase	–	–	–	–	–	–	–
L-Pyrrolydonyl-arylamidase	–	–	–	–	–	+	+
Beta-galactosidase	–	–	–	–	–	–	–
Beta-N-acetyl-glucosaminidase	–	–	–	–	–	–	–
Glutamyl arylamidase pNA	–	–	–	–	–	+	+
Gamma-glutamyl-transferase	+	+	+	+	–	+	+
Beta-glucosidase	–	–	–	–	–	–	–
Beta-xylosidase	–	–	–	–	–	–	–
Beta-alanine arylamidase pNA	–	–	–	–	–	–	–
L-proline-arylamidase	+	+	+	+	+	+	+
Lipase	–	–	–	–	–	–	–
Palatinose	–	–	–	–	–	–	–
Tyrosine arylamidase	+	+	+	+	+	+	+
Urease	–	–	–	–	–	–	+
Alpha-glucosidase	–	–	–	–	–	–	–
Beta-N-acetyl-galactominidase	–	–	–	–	–	–	–
Alpha-galactosidase	–	–	–	–	–	–	–
Phophatase	+	+	+	–	+	+	+
Glycine arylamidase	–	–	–	–	–	–	+
Ornithine decarboxylase	–	–	–	–	–	–	–
Lysine decarboxilase	–	–	–	–	–	–	–
Beta-glucuronidase	–	–	–	–	–	–	–
Glu-Gly-Arg-arylamidase	–	–	–	–	–	–	–
Assimilation of:							
Adonitol	–	–	–	–	–	–	–
L-arabitol	–	–	–	–	–	–	–
D-cellobiose	–	–	–	–	–	–	–
D-glucose	–	–	–	–	–	–	–
D-maltose	–	–	–	–	–	–	–
D-mannitol	–	–	–	–	–	–	–
D-mannose	–	–	–	–	–	–	–
D-sorbitol	–	–	–	–	–	–	–
Saccharose	–	–	–	–	–	–	–
D-tagatose	–	–	–	–	–	–	–
D-trehalose	–	–	–	–	–	–	–
Citrate (sodium)	+	+	+	+	+	+	+
Malonate	+	+	+	+	–	–	–
5-keto-D-gluconate	–	–	–	–	–	–	–
L-histidine	–	+	–	–	–	–	–
Coumarate	–	–	–	–	–	–	–
L-malate	+	+	+	+	–	+	+
Ellman	+	+	+	+	+	+	+

L-lactacte	+	+	+	+	–	+	+
Glucose fermentation	–	–	–	–	–	–	–
O/129 resistance	–	–	–	–	–	–	–

+, positive reaction. –, negative reaction. nd, not determined.
